# mTOR Activation in Liver Tumors Is Associated with Metabolic Syndrome and Non-Alcoholic Steatohepatitis in Both Mouse Models and Humans

**DOI:** 10.3390/cancers10120465

**Published:** 2018-11-22

**Authors:** Takahiro Okuno, Anna Kakehashi, Naomi Ishii, Masaki Fujioka, Min Gi, Hideki Wanibuchi

**Affiliations:** Department of Molecular Pathology, Osaka City University Graduate School of Medicine, Asahi-machi 1-4-3, Abeno-ku, Osaka 545-8585, Japan; m2026860@med.osaka-cu.ac.jp (T.O.); anna@med.osaka-cu.ac.jp (A.K.); m1159070@med.osaka-cu.ac.jp (N.I.); m2066048@med.osaka-cu.ac.jp (M.F.); mwei@med.osaka-cu.ac.jp (M.G.)

**Keywords:** mTOR, NASH, metabolic syndrome, hepatocellular carcinoma, mouse models

## Abstract

Non-alcoholic steatohepatitis (NASH) can cause liver fibrosis and cirrhosis, with final progression to hepatocellular carcinoma (HCC) in some cases. Various factors have been suggested to be involved in the development of NASH. Considering the many possible contributing factors, we postulated that mechanisms of progression from NASH to HCC could differ depending on the risk factors. In the present study, we applied two mouse models of NASH–HCC and performed histopathological and proteome analyses of mouse liver tumors. Furthermore, to compare the mechanisms of NASH–HCC progression in mice and humans, we investigated HCCs in humans with a background of metabolic syndrome and NASH, as well as HCCs associated with hepatitis virus infection by immunohistochemistry. It was demonstrated that upstream regulators associated with the mammalian target of rapamycin (mTOR) pathway were altered in liver tumors of mice with metabolic syndrome characteristics (TSOD mice) using proteome analysis. Immunohistochemical analysis showed that mTOR was characteristically phosphorylated in liver tumors of TSOD mice and HCCs from metabolic syndrome cases in humans. These results indicated that the mTOR pathway is characteristically activated in liver tumors with metabolic syndrome and NASH, unlike liver tumors with other etiologies.

## 1. Introduction

Liver cancer is number five in the world in terms of incidence and second for mortality worldwide, the prognosis generally being poor. Hepatocellular carcinoma (HCC) accounts for 80% of liver cancers, with 70–80% of HCC patients having chronic liver disease including chronic hepatitis and cirrhosis, with hepatitis B virus (HBV) and hepatitis C virus (HCV) infection as the major etiology [1,2]. However, in recent years non-alcoholic steatohepatitis (NASH) has been increasingly implicated as a cause of liver fibrosis and cirrhosis and also HCCs [3,4,5]. NASH is a clinicopathologic syndrome that is not associated with excessive alcoholic intake, but does have histological features similar to alcoholic hepatitis, like fatty deposition, inflammation, and fibrosis. It is directly associated with obesity and metabolic abnormalities such as type 2 diabetes and dyslipidemia [6,7,8]. With the rising rates of obesity and metabolic syndrome, NASH is a growing health problem accounting for an increasing proportion of liver cancers [6,7,8]. However, the natural course of NASH–HCC progression and the difference between HCCs related to NASH and those with other etiologies like alcohol and hepatitis virus are still not fully understood. Recent studies have reported that HCC can arise in NASH patients without cirrhosis, in contrast to HCCs with other risk factors developed in cirrhotic liver [9,10]. These results indicate that the degree of fibrosis in the background liver may affect the HCC development and that the mechanisms of HCC development with NASH may differ from those with other etiologies.

The initial theory for the pathogenesis of NASH assumed a “two-hit hypothesis” [11]. It was suggested that triglyceride accumulation, or steatosis, increased hepatic susceptibility to a “second hit” related to rise in inflammatory cytokines and/or adipokines, mitochondrial dysfunction and elevated oxidative stress, which all promote steatohepatitis and fibrosis. Various studies have indicated that changes in serum cytokines [12,13,14], microflora of the gut and bile duct [15,16,17], and endoplasmic reticulum stress [18] may additionally contribute. Our recent results pointed to significant activation of β-catenin, Nrf2, SMAD3-TGF-β, SREBP-LXRα and NRIP1, and inhibition of PPARs and p53 in human NASH and/or HCC biopsies [19]. Therefore, the multiple parallel hit hypothesis has been proposed and adapted recently, suggesting that inflammation might lead to steatosis in NASH and various processes including inflammatory mediators from the gut and adipose tissues might act parallelly in the development of NASH [20]. However, the mechanisms of progression from NASH to HCC remain to be fully elucidated. 

Various genetic and environmental factors have been suggested to be involved in the development of NASH. For example, individuals with a variant of transmembrane 6 superfamily member 2 gene (*TM6SF2*) exhibit increased liver fat but reduced levels of circulating lipids, representing a metabolically silent variant of NASH [21]. Furthermore, some drugs, intestinal and metabolic diseases are reported to cause NASH in humans [22,23,24,25,26,27,28]. Several animal models of NASH-associated HCC are available for investigation of the mechanisms of carcinogenesis. For instance, methionine- and choline-deficient diet (MCDD) NASH models have been developed [29]. As methionine and choline are important for the production of very low-density lipoprotein (VLDL), their deficiency leads to extensive hepatic lipid accumulation and steatohepatitis, but obesity and other features of metabolic syndrome are lacking. To overcome this problem, choline-deficient high-fat diet (CHFD) [30] and choline-deficient, L-amino acid-defined, high-fat diet (CDAHFD) [31] models have been developed, but these treatments do not always lead to obesity and metabolic syndrome in animals. In another model, Tsumura Suzuki obese diabetes (TSOD) mice of a ddy strain were developed with obesity and diabetes, leading to NASH, arising spontaneously due to overeating, but the genetic background and mechanisms of induction are not fully understood [32,33]. Genetically engineered farnesoid X receptor (FXR) knockout mice [34,35] and melanocortin 4 receptor (Mc4r) knockout mice [36], and models in which NASH and liver tumors are induced in mice by drugs such as streptozotocin [37], have been also documented.

Considering the apparent differences in the development of NASH and its progression to HCC in humans and animals, despite the shared major contributions of obesity and metabolic syndrome, we postulated that mechanisms of progression from NASH to HCC could differ depending on the risk factors. To address this question and investigate the mechanisms, here we applied two mouse models of NASH–HCC (TSOD- and CDAHFD-fed mouse models) and performed histopathological and proteome analyses of mouse livers, searching for altered signaling pathways and upstream regulators by Ingenuity Pathway Analysis (IPA) and immunohistochemically verified target proteins. B6C3F1 mice given diethylnitrosamine (DEN) and phenobarbital (PB) in a two-step hepatocarcinogenesis bioassay [38] served as secondary experimental controls. Comparing the results, we found that the mammalian target of rapamycin (mTOR) pathway was characteristically activated in liver tumors of TSOD mice. To compare the results of mouse models and humans and determine the differences in the mechanism of tumor development with NASH and virus infection, we also assessed the state of mTOR activation in HCCs of patients with metabolic syndrome and NASH as well as examples associated with hepatitis virus infection. It was revealed that the mTOR pathway is also characteristically activated in human HCCs associated with metabolic syndrome and NASH. 

## 2. Results

### 2.1. General Conditions and Pathological Findings of Mouse Models

Data on general conditions and organ weights of TSOD and 45% CDAHFD-fed NASH model mice are summarized in Table 1. Significant increases of final body weights, absolute and relative liver weights, and blood glucose levels were found in TSOD mice as compared to the control TSNO mice. In C57BL/6J mice fed CDAHFD, a significant decrease of final body weights, and increases of absolute and relative liver weights were detected, relative to the control animals administered the control diet. Blood glucose levels of C57BL/6J mice on CDAHFD and control diet did not differ.

These results indicate that obesity and diabetes associated with metabolic syndrome developed in the TSOD mice as previously reported [31,32], but not in CDAHFD-treated C57BL/6J animals.

**Figure 1:**
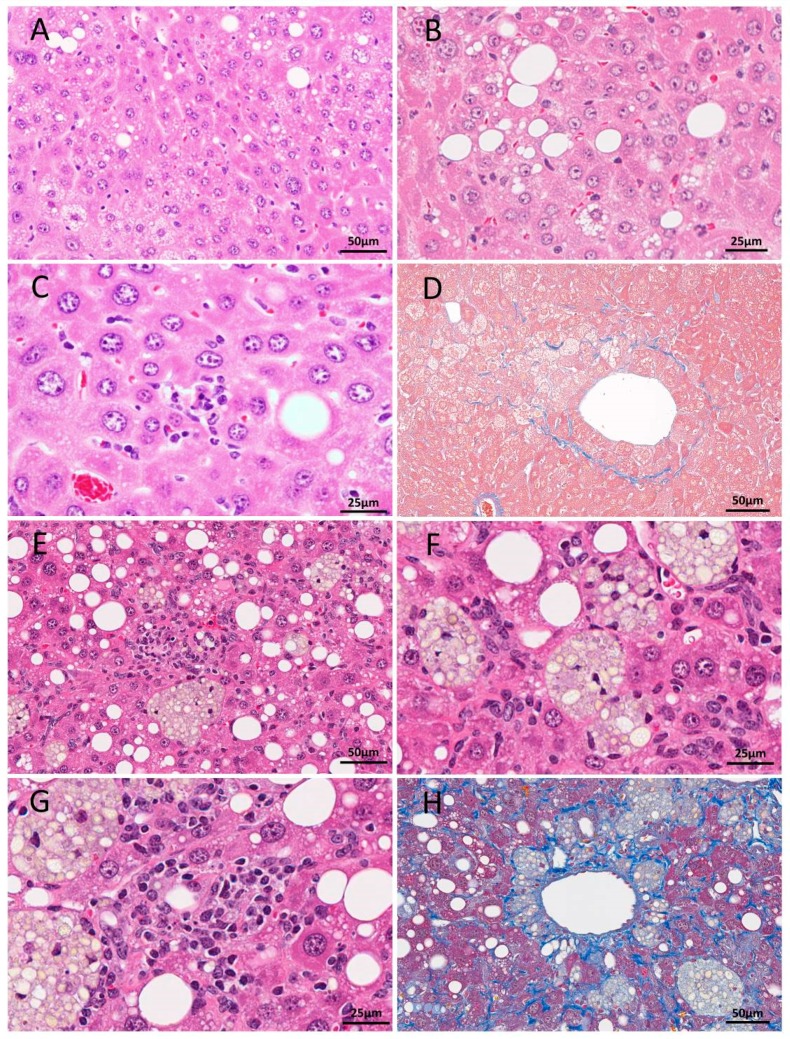
H&E (**A**–**C**) and Azan (**D**) staining of liver sections from 52-week-old TSOD mice and H&E (**E**–**G**) and Masson trichrome (**H**) staining of livers in a 48-week-old mouse fed CDAHFD. In the TSOD mice livers, mild fatty change and ballooning (**A**,**B**), mild inflammation (**C**) and Zone 3 perisinusoidal fibrosis (**D**) were detected. Fatty change, lipogranuloma (**E**,**F**), severe inflammation (**G**) and severe fibrosis (**H**) were obvious in CDAHFD-fed C56Bl/6J mice. Scale bar: 25 μm (**B**,**C**,**F**,**G**), 50 μm (**A**,**D**,**E**,**H**).

Histopathological findings for mouse livers in both NASH models are summarized in Table 2. Significantly increased incidence and/or multiplicities of total liver tumors (hepatocellular carcinomas (HCCs) and hepatocellular adenomas (HCAs)) were detected in both TSOD and CDAHFD-administered mice. The detailed classifications of liver tumors of both model mice are shown in Table S1. Pathological findings reflecting NASH development, including fatty degeneration, inflammatory cell infiltration, hepatocellular ballooning and perivenous fibrosis were observed in both TSOD and CDAHFD-treated animals (Figure 1). The degree of steatosis, inflammation, and fibrosis was more severe in the CDAHFD case.

### 2.2. Protein Expression in Liver Tumors of TSOD Mice Identified by QSTAR Elite LC-MS/MS

To identify proteins associated with the development of liver tumors in NASH, a combination of QSTAR Elite LC-MS/MS, ProteinPilot and Ingenuity Pathway Analysis (IPA) analyses was performed. Proteins differentially expressed in the liver tumors of TSOD mice in comparison with control TSNO mice livers are listed in Table 3 (99% confidence cut off limit; *p* < 0.05, expression changes greater than 2-fold or lower than 0.5-fold).

Significant upregulation of glutathione S-transferase (GST) Mu 1, Mu2, Mu3 (GSTM1, GSTM2, GSTM3), myoglobin (MB), cytochrome P450 (CYP) 2A5 (CYP2A5), apolipoprotein A-I preproprotein (APOA1), glutamine synthetase, ornithine aminotransferase (OAT), protein disulfide-isomerase A6 (PDI-6), annexin A5 (ANXA5), carboxylesterase 5 (CES5), epoxide hydrolase 1 (EPHX1), CYP1A2 and pyruvate kinase L/R (PKLR) were observed in TSOD mouse liver tumors. On the other hand, dihydrolipoamide branched chain transacylase E2 (DBT), glutaryl-CoA dehydrogenase (GCDH), CYP4A12A, ornithine carbamoyltransferase (OTC), glycine N-methyltransferase (GNMT), mitochondrial amidoxime-reducing component 1 (MARC1), argininosuccinate synthase (ASS), tetratricopeptide repeat protein 36 (TTC36), 4-aminobutyrate aminotransferase (ABAT), acyl-coenzyme A oxidase 1 (ACOX1), carboxylesterase 1E.6 (CES1E.6), carbamoyl-phosphate synthetase 2, aspartate transcarbamylase and dihydroorotase (CAD), betaine-homocysteine S-methyltransferase 1 (BHMT1), aldehyde dehydrogenase 1 family member L1 (ALDH1L1), sulfotransferase family 4A member 1 (SULT4A1) and major urinary protein 2 (MUP2) were downregulated.

### 2.3. Canonical Pathway and Upstream Regulator Analysis by IPA

Results of IPA upstream regulator analysis predicted activation of insulin like growth factor 1 (IGF-1), rapamycin-insensitive companion of mTOR (RICTOR), nuclear factor, erythroid 2 like 2 (NFE2L2, Nrf2), TNF receptor associated protein 1 (TRAP1), hepatocyte nuclear factor 1 homeobox A (HNF1A), tumor necrosis factor (TNF), nuclear receptor subfamily 1 group I member 2 (NR1I2), interleukin 10 receptor subunit alpha (IL10RA) and versican (VCAN) (z score >2), while inhibition of protein kinase AMP-activated catalytic subunit alpha 1, 2 (PRKAA1, 2), interleukin 4 (IL4); Wilms tumor 1 (WT1) (z score <−2) was found in the liver tumors of TSOD mice relative to control liver tissues of TSNO mice (Table 4).

### 2.4. Immunohistochemical Assessment of mTOR, FOXO3A and S6 in TSOD Mice

**Figure 2:**
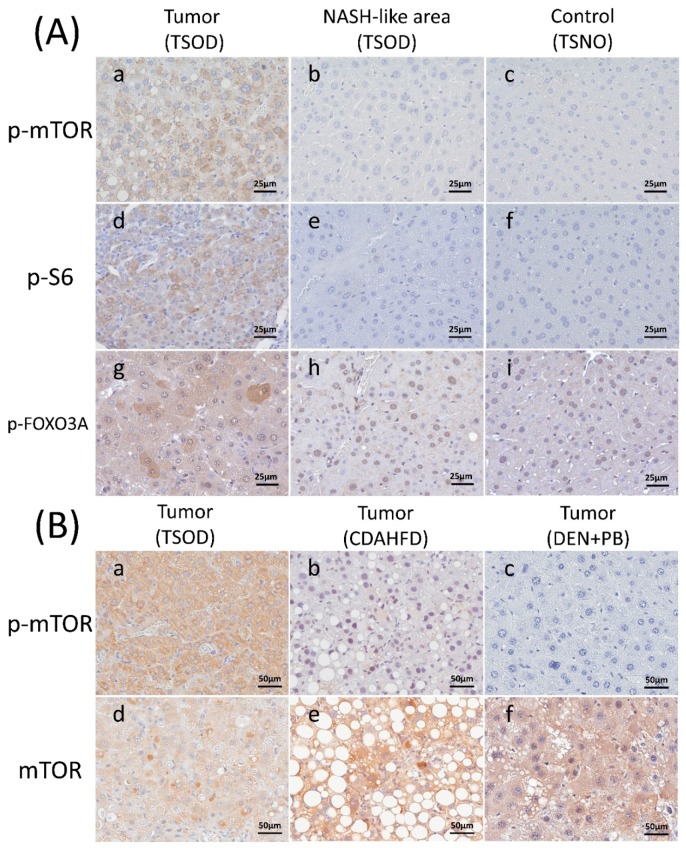
Representative immunohistochemical findings of NASH model mice. (**A**) p-mTOR, p-S6 and p-FOXO3A in liver tumors and liver tissues of TSOD mice and liver tissues of TSNO mice. Immunohistochemistry of p-mTOR in the liver tumor of TSOD mouse (**a**), surrounding NASH-like area of TSOD mouse (**b**) and control normal liver tissue of TSNO mouse (**c**), p-S6 in the liver tumor of TSOD mouse (**d**), surrounding liver tissue of TSOD mouse (**e**) and control normal liver tissue of TSNO mouse (**f**) and p-FOXO3A in the liver tumor of TSOD mouse (**g**), surrounding liver tissue of TSOD mouse (**h**) and control normal liver tissue of TSNO mouse (**i**). Note that p-mTOR, p-S6, and p-FOXO3A are strongly expressed in liver tumors of TSOD mice. (**B**) mTOR and p-mTOR in liver tumors of TSOD, CDAHFD-fed C57BL/6J mice and B6C3F1 mice given diethylnitrosamine (DEN) and phenobarbital (PB). Immunohistochemistry of p-mTOR in liver tumors of TSOD mice (**a**), mice given CDAHFD (**b**) and mice given DEN and PB (**c**) and mTOR in liver tumors of TSOD mice (**d**), mice given CDAHFD (**e**) and mice given DEN and PB (**f**). Note that p-mTOR was characteristically expressed in liver tumors of TSOD mice. Scale bar: 25 μm (**A** (**a**–**i**)), 50 μm (**B** (**a**–**f**)). NASH: Non-alcoholic steatohepatitis; DEN: diethylnitrosamine; PB: phenobarbital; TSOD: Tsumura Suzuki obese diabetes; TSNO: Tsumura Suzuki non-obesity; CDAHFD: choline-deficient, L-amino acid-defined, high-fat diet.

As the IPA pathway and upstream regulator analysis of differential protein expression in TSOD mouse liver tumors demonstrated activation of insulin like growth factor 1 (IGF-1) and RICTOR, we presumed that this could be due to the activation of the mTOR pathway. We therefore performed immunohistochemical assessments of mTOR, phospho-mTOR (p-mTOR), S6, phospho-S6 (p-S6), mice and normal livers of TSNO mice. Staining patterns did not differ greatly between hepatocellular carcinomas (HCCs) and hepatocellular adenomas (HCAs). Therefore, results of IHC examination were combined for HCCs and HCAs (“liver tumors group”). Representative immunohistochemical staining of p-mTOR, S6, and p-FOXO3A is shown in Figure 2A, and results are summarized in Table 5.

Diffuse expression of mTOR and S6 was observed in almost all of liver tumors and surrounding liver tissues (NASH area) of TSOD mice and liver tissues of TSNO mice. On the other hand, p-mTOR and p-S6 were strongly expressed in almost half of TSOD mice liver tumors but not in TSOD liver tissues and TSNO liver tissues and the rate of p-mTOR and p-S6 expression was significantly increased in liver tumors of TSOD mice compared to control liver tissues of TSNO mice (*p* < 0.05). Nuclear FOXO3A overexpression was found in the liver tumors and surrounding tissues of TSOD mice and liver tissues of TSNO mice, being negative in the cytoplasm of tumors and surrounding areas of TSOD mice and liver tissues of TSNO mice. On the other hand, p-FOXO3A cytoplasmic expression was strong in 49% of TSOD mice tumors and only in 15% and 0% of adjacent tissues of TSOD mice and liver tissues of TSNO mice, respectively. The rate of p-FOXO3A cytoplasmic expression was significantly increased in liver tumors of TSOD mice compared to control liver tissues of TSNO mice (*p* < 0.05).

### 2.5. Immunohistochemical Evaluation of mTOR in Liver Tumors of C57BL/6J Mice Fed CDAHFD and B6C3F1 Mice Given DEN and PB

To identify differences in the state of mTOR activity in liver tumors between TSOD and other model mice, we examined mTOR and p-mTOR in tumors of TSOD, CDAHFD-treated C57BL/6J and DEN and PB treated B6C3F1 mice. Representative immunohistochemical findings of mTOR and p-mTOR are shown in Figure 2B and results are summarized in Table 6. 

Diffuse overexpression of mTOR was observed in almost all liver tumors of CDAHFD-administered animals and B6C3F1 mice given DEN and PB. However, strong expression of p-mTOR was detected only in 3% of C57BL/6J cases fed CDAHFD and 1% of B6C3F1 mice given DEN and PB. The rate of p-mTOR diffuse expression in liver tumors of TSOD mice was higher than those of mice fed CDAHFD and mice given DEN and PB (49%, 3%, 1%, respectively).

### 2.6. Immunohistochemical Assessment of mTOR in Human HCCs

**Figure 3:**
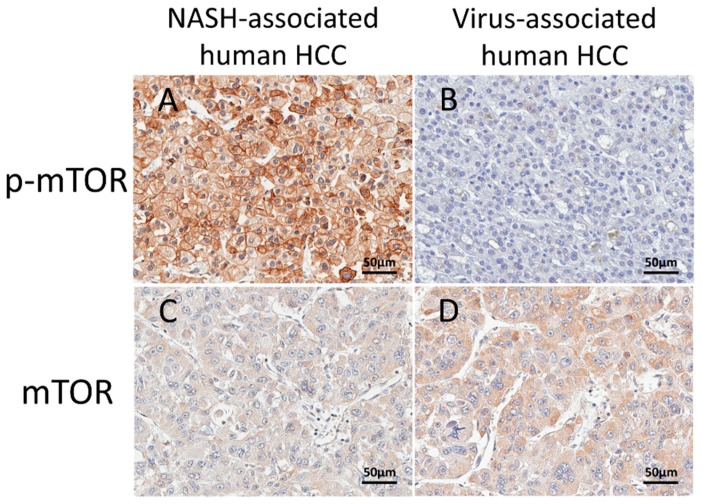
Representative immunohistochemical findings of mTOR and p-mTOR in human metabolic syndrome/NASH-associated and virus-associated HCCs. Immunohistochemistry of p-mTOR in metabolic syndrome/NASH-associated HCC (**A**) and virus-associated HCC (**B**) and mTOR in metabolic syndrome/NASH-associated HCC (**C**) and virus-associated HCC (**D**). Note that p-mTOR was characteristically expressed in metabolic syndrome/NASH-associated HCCs. Scale bar: 50 μm (**A**–**D**). HCC: hepatocellular carcinoma.

To apply the results of NASH mouse models to human HCCs, we performed the immunohistochemical evaluation of mTOR in metabolic syndrome/NASH-associated HCCs and virus-associated HCCs in human. Representative immunohistochemical findings of mTOR and p-mTOR are shown in Figure 3 and results are summarized in Table 6. mTOR was overexpressed in more than half of metabolic syndrome/NASH-associated HCCs and virus-associated HCCs. p-mTOR expression was high in 63% of metabolic syndrome/NASH-associated HCCs, but only in 3% of virus-associated HCCs. The rate of strong p-mTOR expression was much higher in metabolic syndrome/NASH-associated HCCs than that in virus-associated HCCs.

## 3. Discussion

In the present study, we revealed upstream regulators associated with the mTOR pathway to be altered in liver tumors of NASH model mice with metabolic syndrome (TSOD mice) using QSTAR Elite LC-MS/MS and IPA analyses. Immunohistochemical examination of TSOD mice revealed that mTOR, S6 and FOXO3A were phosphorylated in liver tumors of TSOD mice, supporting the results of QSTAR Elite LC-MS/MS and IPA analyses. Immunohistochemistry of liver tumors in three mice models and human HCCs showed phosphorylation of mTOR to only be characteristic in TSOD mice and metabolic syndrome/NASH-associated lesions. These results suggest that mTOR pathway activation is characteristic in liver tumors associated with metabolic syndrome and NASH in both mice and humans. 

Comparing the two NASH model mice (TSOD mice and C57BL/6J mice fed 45% CDAHFD), both showed NASH-like histology in their liver tissues including fatty degeneration, inflammatory cell infiltration, fibrosis, and tumor development, but the severity was greater in the TSOD case featuring overexpression of p-mTOR. The differences may reflect involvement of numerous factors in NASH development [6,7,8,22,23,24,25,26,27,28] or indicate that NASH itself may be a heterogeneous syndrome. 

mTOR is a serine/threonine protein kinase involved in various processes involved in cell growth and survival, metabolism and angiogenesis [39]. mTOR signaling is mediated by two distinct mTOR complexes named mTOR complex 1 (mTORC1) and mTOR complex 2 (mTORC2) [39]. mTORC1 is reported to phosphorylate S6, and promote cell proliferation, protein synthesis and lipogenesis [39]. Furthermore, its activation is influenced by energy status, oxygen levels, proinflammatory cytokines, and growth factors, including IGF-1 via PI3K-AKT signaling [39]. Activation of the PI3K-AKT pathway also induces phosphorylation of FOXO3a followed by consequent inhibition its transcriptional activity, finally resulting in suppression of apoptosis. In the present study, QSTAR Elite LC-MS/MS and IPA analyses predicted activation of IGF-1 and immunohistochemical staining showed phosphorylation of mTOR, S6 and FOXO3a in the liver tumors of TSOD mice. These results suggest that mTOR pathway is activated via IGF signaling in liver tumors associated with NASH and metabolic syndrome and resultant promotion of cellular proliferation and suppression of apoptosis may contribute to tumorigenesis. Drugs inhibiting mTOR have been reported to improve insulin resistance and NASH in rats with diabetes [40,41]. Further long-term studies using NASH model mice with mTOR inhibitors are needed to confirm the postulated relationship between activated mTOR and NASH–HCC progression and elucidate underlying mechanisms. Furthermore, recent studies reported that mTOR pathway was activated in hepatic cell lines due to the HCV infection in vitro [42] and drugs targeting mTOR pathway inhibit fibrosis in rat livers treated with carbon tetrachloride (CCL_4_) [43,44], indicating the potential association of mTOR pathway with the risk factors of HCCs other than NASH inducing fibrosis. However, in the present study, p-mTOR was not strongly expressed in NASH-like area of livers with fibrosis in both TSOD mice and mice fed CDAHFD. Further studies to determine which factor is closely related to the activation of mTOR pathway and examine the association between mTOR pathway and the fibrotic process of the liver are needed to confirm the specificity of mTOR pathway activation in liver tumors with NASH and metabolic syndrome.

The mTOR pathway has been reported to be activated in 15–50% of human HCCs [45,46,47]. However, these studies did not consider the underlying etiology or include NASH and metabolic syndrome factors as causes of HCCs. mTOR inhibitors have found application in treatment for various malignancies like renal cell carcinomas [48,49,50] and breast cancers [51,52,53]. Disappointingly, Everolimus, one mTOR inhibitor, did not increase the overall survival in patients with advanced HCC who were resistant to sorafenib in a first clinical trial [54]. However, the etiology of HCC was not focused on and the proportion associated with NASH was only 3%. Considering that activation of the mTOR pathway appears characteristic of HCCs associated with metabolic syndrome unlike HCCs with HBV and HCV infection, future clinical studies should take this into account.

As described above, mediators released from adipose tissues play a key role in the pathogenesis of NASH. Adiponectin, one of the adipokines, contributes to the metabolism of fat and glucose and is related to type 2 diabetes and NASH [20,55]. Indeed, serum adiponectin was decreased in patients with NASH [56] and drugs that increase the serum adiponectin levels improve the inflammation and fibrosis in the liver of NASH [57]. Furthermore, previous studies shown that low adiponectin levels were related to the risk for various cancers [58,59]. It was also reported that adiponectin decreased the cellular proliferation and induced apoptosis through the inhabitation of mTOR pathway [60,61]. Considering these facts, adiponectin may play an important role in the activation of mTOR pathway observed in liver tumors of TSOD mice and patients with NASH and metabolic syndrome. IGF-1 is upstream of mTOR and our results of IPA analysis in TSOD mice demonstrated its activation. However, serum and liver IGF-1 levels were found to be decreased in diabetic rats [62] and serum IGF is reduced in patients with NASH [63]. Thus, IGF signaling may be dependent on complex mechanisms acting separately in liver tumors with a background of metabolic syndrome and NASH and in serum. Our IPA analysis also showed inhibition of PRKAA1 and PRKAA2, AMP-activated protein kinases (AMPKs) that play roles in cellular energy control. AMPK activation occurs with by stresses that inhibit ATP production such as hypoxia and hypoglycemia and may downregulate mTOR signaling and cellular proliferation [63,64]. AMPK is related to metabolic diseases including diabetes [65,66] and drugs such as metformin cause its activation [64,65,66]. AMPK activators also can reduce the risk of cancer development in humans [67,68] and prevent tumorigenesis in mice [69,70]. The fact that AMPK was downregulated in the liver tumors of TSOD mice indicates this might underly the upregulation observed for mTOR. Thus, AMPK activators might become useful tools for preventing progression from NASH to HCC. 

Proteome analyses have revealed that various proteins including enzymes were upregulated or downregulated in liver tumors in TSOD mice. Dysregulation of CYPs was reported in human HCC [71] and NASH [72] and upregulation of CYP 2A5 and CYP 1A2 was noted in the livers of TSOD mice in the present study. CYP1A is reported to be associated with susceptibility to tumor development in mice [73]. Takahashi et al. revealed that glutamine synthetase (GS) is overexpressed in more than 70% of liver tumors in TSOD mice [74], and it has also been proposed as a useful marker for the differential diagnosis of early HCC from benign hepatocellular lesions in human [75]. Our results of proteome analyses are consistent with GS playing a similar role in distinguishing early HCC associated with NASH. Overexpression of ornithine aminotransferase (OAT), a key enzyme that converts ornithine to glutamate, was observed in HCCs of *Psammomys obesus* [76]. Since an OAT inactivator was found to suppress proliferation in HCC cell lines [76], the present demonstration of upregulation in liver tumors of TSOD mice implies a role in carcinogenesis. Glycine N-methyltransferase (GNMT) is an enzyme that plays an important role in regulating the ratio between S-adenosylmethionine (SAM) and S-adenosylhomocysteine (SAH) [77,78]. GNMT-knockout mice develop liver steatosis, fibrosis, and HCCs [77,78] and show high susceptibility to aflatoxin B1, a liver carcinogen [79]. Our proteome analysis finding of downregulation of GNMT in the liver tumors of TSOD mice is in line with GNMT being considered to suppress HCC development. Annexin A1 is reported to be a biomarker predicting a poor prognosis of HCC [80], like annexin A2 [81] and carboxylesterase (CES1) [82,83]. Altered expression of annexin A5, CES5 and CES6 in the present study may indicate that these proteins could also be useful in predicting the outcome of HCCs associated with NASH. Mice knocked out for betaine–homocysteine S-methyltransferase (BMHT), an enzyme that converts homocysteine to methionine, demonstrate fatty change of the liver and HCC due to disturbance of choline metabolism decreasing the production of VLDL [84]. In the present study, the expression of BMHT in the liver tumors of TSOD was downregulated, which might contribute to the disturbance of VLDL production and the accumulation of fat in the liver.

## 4. Materials and Methods 

### 4.1. Animals, Experimental Design, and Treatments

**Figure 4:**
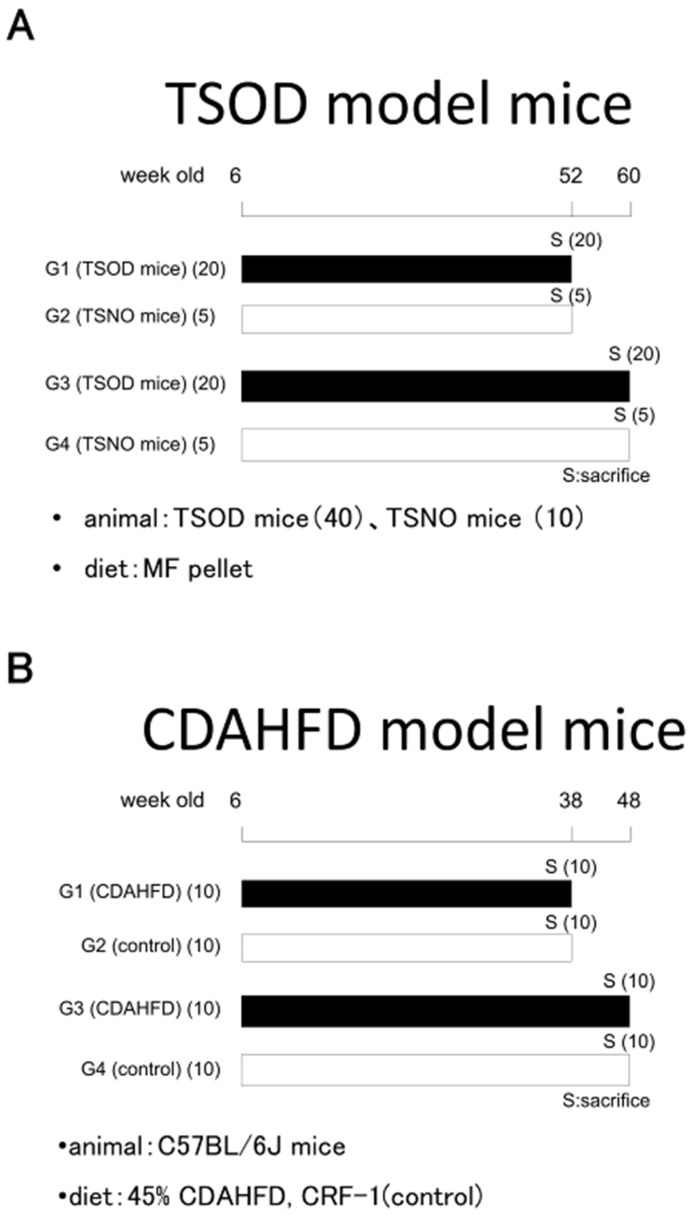
Experimental protocols. (**A**) TSOD NASH model mice with metabolic syndrome; (**B**) NASH model C57Bl/6J mice administered 45% CDAHFD.

A total of 40 six-week-old Tsumura Suzuki obese diabetes (TSOD) mice, hereafter referred to as NASH model mice with metabolic syndrome, were fed a standard diet for 46 or 54 weeks. As controls, 10 six-week-old Tsumura Suzuki non-obesity (TSNO) mice (SLC, Sizuoka, Japan), of the same ddy strain, were also fed a standard diet for the same periods (Figure 4A). In the dietary NASH model, 20 six-week-old C57BL/6J mice were administered a choline-deficient, L-amino acid-defined, 45 kcal% high-fat diet (45% CDAHFD) containing 0.1% methionine (Charles River, Kanagawa, Japan) for 32 or 42 weeks, with 20 further animals given a control diet (CRF-1, Oriental Yeast Co., Ltd., Tokyo, Japan) (Figure 4B). Animals were euthanized under isoflurane at the end of the experimental periods. Body weights and fasting blood glucose levels were measured before autopsy and a macroscopic examination was immediately performed. Thereafter, mouse liver tissues and tumors were fixed in 10% buffered formalin and prepared for routine histology (hematoxylin and eosin (H&E) staining), collagen fiber staining (Masson trichrome and Azan stains) and immunohistochemistry, or frozen in liquid nitrogen for biochemical and proteome analyses. Liver tumors occurring in B6C3F1 mice given diethylnitrosamine (DEN) and phenobarbital (PB) in a two-step hepatocarcinogenesis bioassay obtained from a previous experiment [38], were also included for immunohistochemical analysis for comparison. All the experimental procedures were approved by the Ethics Committee of the Institutional Animal Care and Use Committee of Osaka City University Graduate School of Medicine (14011, 3/9/2014).

### 4.2. Human HCC Samples, University Review Board Approval, and Informed Consent 

A total of 77 patients who had undergone resection of histopathologically proven HCC at Osaka City University hospital from 2009 to 2017 were enrolled in the study. Those for whom NASH was histologically confirmed by biopsy were few, because clinically typical NASH cases are not usually biopsied, and the diagnosis of NASH can be difficult in resected specimens of HCCs because the characteristic histological findings may be unclear once fibrosis fully progresses. Therefore, in the present study, 46 HCC patients with complications or a history of obesity, diabetes, dyslipidemia, hypertension, or NASH and without hepatitis virus infection or excessive alcohol consumption were considered as metabolic syndrome/NASH-associated HCC cases. Thirty-one HCC patients with a background of hepatitis B or C virus infection and without history of obesity, diabetes, dyslipidemia, hypertension, NASH or alcohol drinking were considered the virus-associated group. Clinicopathological features of the enrolled patients were obtained from medical records (Table S2). We carefully reviewed the histopathological samples of all cases and chose representative blocks for immunohistochemistry. The human study was approved by the Ethics Committee of Osaka City University Graduate School of Medicine (Osaka, Japan) and conducted in accordance with the principles of the declaration of Helsinki (4044, 26/4/2018).

### 4.3. Proteome Analyses of NASH Model Mouse Livers and Tumors

Proteome analyses of liver and tumor samples from TSOD and TSNO mice were performed with the DiNa-AI nano LC System (KYA Technologies, Tokyo, Japan) coupled to a QSTAR Elite hybrid mass spectrometer (AB Sciex, Concord, Ontario, Canada) through a NanoSpray ion source (AB Sciex), as previously described [85]. Samples were prepared using T-PER™ Tissue Protein Extraction Reagent (Thermo Fisher Scientific, Tokyo, Japan). For quantitative MS/MS analysis, labeling was achieved with 4-plex iTRAQ isobaric reagents [85] for the following: in the TSOD mouse NASH model with metabolic syndrome, 115 liver tumors and 116 adjacent non-tumor livers (NASH area) of TSOD mice as well as 117 normal liver tissues of TSNO mice. iTRAQ-labeled samples were refined by cation exchange column chromatography. Peptides were eluted as six fractions (1 mL of KCl solution of 10, 50, 70, 100, 200, and 350 mM), and the supernatants were vaporized in a vacuum centrifuge. Samples were demineralized and concentrated using a Sep-Pak column (C18 Plus Light Cartridge, Waters, MA, USA), evaporated in a vacuum centrifuge, dissolved in 20 μL of 0.1% formic acid, and applied to QSTAR Elite LC-MS/MS. Each sample was run for 150 min, with MS/MS data analyzed using Swiss Protein Database (HUMAN) and ProteinPilot Software 2.0 (AB Sciex, Tokyo, Japan). Results were analyzed using a 95% confidence cutoff limit. Protein levels were quantified by comparing iTRAQ reporter ion intensities between liver tumors and adjacent liver tissues of NASH area with normal liver tissue values. Protein ratios at *p* < 0.05 were considered significant. To investigate the mechanisms of liver tumor induction in NASH model mice, proteins detected by QSTAR Elite LC-MS/MS were subjected to Ingenuity Pathway Analysis (IPA) (Ingenuity Systems, Mountain View, CA, USA) for identification of potential candidate biomarkers. Altered upstream regulators were assessed in liver tumors of NASH model mice compared to relevant controls. As with IPA analysis, z-scores above or lower than 2 were considered significant.

### 4.4. Immunohistochemistry and Scoring for Livers of NASH Model Mice and Human HCCs 

To determine the state of mTOR pathway activation in liver tumors of TSOD mice, antibodies against mTOR (dilution 1:100; Cell Signaling, Danvers, MA, USA), phospho-mTOR (p-mTOR) (dilution 1:1000; Abcam, Cambridge, UK), forkhead box O3A (FOXO3a) (dilution 1:1000; Cell Signaling), phospho-FOXO3a (p-FOXO3a) (dilution 1:100; Abcam, Cambridge, UK), ribosomal protein S6 kinase (S6) (dilution 1:100; Cell Signaling) and phospho-S6 (p-S6) (dilution 1:400; Cell Signaling) were applied for immunohistochemistry (IHC) with formalin-fixed paraffin-embedded (FFPE) tissue sections and a standard ABC method. After deparaffinization followed by gradual dehydration and antigen retrieval (98 °C incubation in citric acid buffer for 20 min), sections were incubated with 0.3% hydrogen peroxide for 5 min to inactivate endogenous peroxidase activity. Exposure to primary antibodies was overnight at 4 °C. Peroxidase reactions were developed using 3,3-diaminobenzidine tetrahydrochloride (DAB). Hematoxylin was used for counterstaining. 

To examine differences in the state of mTOR pathway activation according to background factors, mTOR and p-mTOR immunostaining was also conducted with liver tumors from mice fed 45% CDAHFD or treated with DEN and PB. Similarly, human HCCs were examined. Stained sections were evaluated by two pathologists and the results of immunohistochemistry were graded from score 0 to 3+, as follows: score 0, negative staining in all cells; score 1+, positive staining in <25% of hepatocytes; score 2+, positive staining covering 25–50% cells; and score 3+, positive staining in >50% cells.

### 4.5. Statistical Analysis

The data were subjected to statistical analysis using the StatLight-2000(C) program (Yukms, Kanagawa, Japan). Results were calculated as mean ± standard deviation (SD) values. Student’s *t*-test, Mann‒Whitney U-test (two-sided), or Fisher exact test was used for comparison between groups. Immunohistochemical staining was evaluated using χ^2^ test, with *p* values < 0.05 considered statistically significant.

## 5. Conclusions

**Figure 5:**
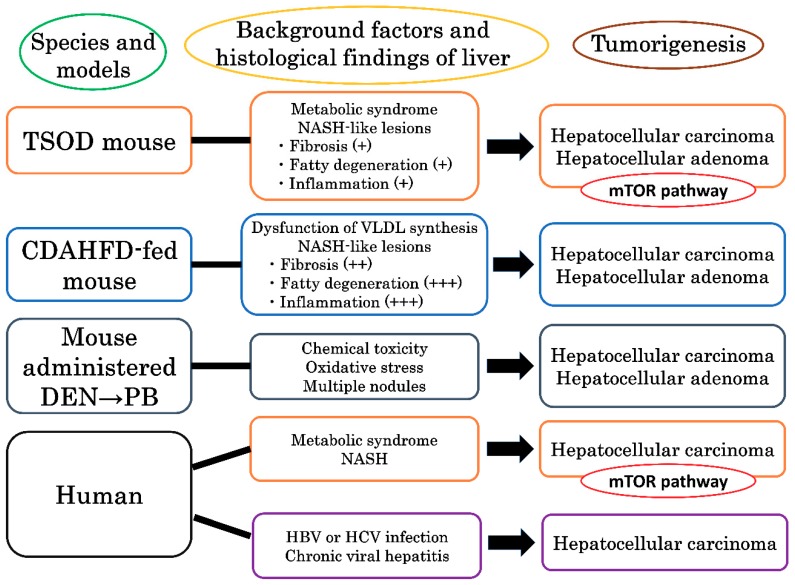
The mTOR pathway is characteristically activated in liver tumors associated with metabolic syndrome and NASH in both mice and humans.

In conclusion, in the present study we demonstrated that the mTOR pathway is characteristically activated in liver tumors associated with metabolic syndrome and NASH, unlike liver tumors with other etiologies (Figure 5). From the results, the mTOR pathway appears to be associated with progression from NASH to HCC, and mTOR inhibitors may be effective for treatment of NASH–associated HCCs.

## Figures and Tables

**Table 1:** Final body and organ weights in two mouse models.

Model	TSOD Model				CDAHFD Model			
Age	52-weeks		60-weeks		38-weeks		48-weeks	
Mice/treatment	TSNO	TSOD	TSNO	TSOD	Control	CDAHFD	Control	CDAHFD
No. mice ^a^	5	18	5	16	10	10	8	10
Final body weight (g)	39.9 ± 3.7	61.6 ± 3.8 *	40.6 ± 3.7	64.0 ± 2.7 *	32.1 ± 2.3	25.5 ± 1.8 *	35.3 ± 2.3	26.1 ± 2.2 *
Absolute liver weight (g)	1.4 ± 0.2	2.6 ± 0.3 *	1.4 ± 0.1	3.0 ± 0.6 *	1.2 ± 0.1	1.7 ± 0.2 *	1.4 ± 0.1	2.0 ± 0.3 *
Relative liver weight (%)	3.6 ± 0.1	4.2 ± 0.4 *	3.6 ± 0.2	4.6 ± 0.8 *	3.8 ± 0.2	6.9 ± 1.3 *	4.1 ± 0.1	7.9 ± 1.3 *
Blood glucose (g/dL)	91 ± 19	112 ± 35	98 ± 12	131 ± 46 *	81 ± 41	62 ± 10	70 ± 8	72 ± 13

* *p* < 0.05, compared to the respective controls; ^a^ Final number of mice that survived. TSOD: Tsumura Suzuki obese diabetes; TSNO: Tsumura Suzuki non-obesity; CDAHFD: choline-deficient, L-amino acid-defined, high-fat diet.

**Table 2:** Histological findings in mouse models.

Model	TSOD Model				CDAHFD Model			
Age	52-weeks		60-weeks		38-weeks		48-weeks	
Mice/treatment	TSNO	TSOD	TSNO	TSOD	control	CDAHFD	control	CDAHFD
No. of animals ^a^	5	18	5	16	10	10	8	10
Liver tumors (HCC + HCA)								
Incidence (%)	1 (20)	11 (61)	0 (0)	13 (81) *	0 (0)	4 (40) *	0 (0)	8 (80) *
Multiplicity (No./mouse)	0.2 ± 0.4	1.0 ± 1.0	0.0 ± 0.0	1.8 ± 1.3 *	0.0 ± 0.0	1.2 ± 1.9	0.0 ± 0.0	2.3 ± 0.9 *
NASH liver tissues ^b^								
Steatosis	-	+	-	+	-	+++	-	+++
Lobular inflammation	-	+	-	+	-	+++	-	+++
Hepatocyte ballooning	-	+	-	+	-	+	-	+
Fibrosis	-	+	-	+	-	++	-	++

HCC, hepatocellular carcinoma; HCA, hepatocellular adenoma. * *p* < 0.05, as compared to the control. ^a^ Final number of survived animals subjected to the analysis; ^b^ NASH liver tissue was graded using NASH activity score and NASH fibrosis stage as described below and most frequent scores were shown. Steatosis: amount of area involved by steatosis; -, <5%, +, 5–33%; ++, <33–66%; +++, >66%. Lobular inflammation: number of inflammatory cell foci per a field of view at ×200 magnification; -, no foci; +, <2 foci; ++, 2–4 foci; +++, >4 foci. Hepatocellular ballooning: the existence of ballooned hepatocytes; -, no ballooned hepatocytes; +, rare but definite ballooned hepatocytes or diagnostically borderline cases; ++, cases with prominent ballooning hepatocytes. Fibrosis: the degree of fibrosis; -, none; +, Zone 3 perisinusoidal fibrosis; ++, Zone 3 perisinusoidal and portal/periportal fibrosis; +++, bridging fibrosis; ++++, cirrhosis; NASH, Non-alcoholic steatohepatitis.

**Table 3:** Differentially-expressed proteins in the liver tumors of TSOD mice detected by QSTAR Elite LC/MS/MS.

Protein	GI Number	log Fold Change	*p* Value	Location	Function
Glutathione S-transferase Mu 3 (GSTM3)	33468899	3.2771	0.0011	C, CB	GM, XM
Myoglobin (MB)	255708425	3.2034	0.0000	C	BFCD, MO, CP,
Cytochrome P450 2A5 (CYP2A5)	75832129	2.9672	0.0001	C, CMT, EPR	ORP, CP, XM
Apolipoprotein A-I preproprotein (APOA1)	160333304	2.2397	0.0000	C, ES, N, EPR	LM, LT, LS
Glutamine synthetase (GLNS)	31982332	2.0976	0.0000	C, N, EPR	GBP, PRIS, CP
Ornithine aminotransferase (OAT)	8393866	1.7858	0.0000	C, MM	OM, AAM
Protein disulfide-isomerase A6 (PDI-6)	377833208	1.4671	0.0000	C, ES, EPR	ORP, PF, RERS
Glutathione S-transferase Mu 2 (GSTM2)	6680121	1.4044	0.0000	C, ES	GM, XM
Annexin A5 (ANXA5)	6753060	1.3512	0.0000	C, ES	RCI, BC
Carboxylesterase 2E (CES2E)	27370126	1.3235	0.0000	C, EPR, MS	XM
Glutathione S-transferase Mu 5 (GSTM5)	6754084	1.2416	0.0000	C	GM, XM
Epoxide hydrolase 1 (EPHX1)	6753762	1.0670	0.0000	EPR, PM	LM, XM
Cytochrome P450 1A2 (CYP1A2)	6753566	1.0183	0.0000	EPR, MS	ORP, XM, CR
Pyruvate kinase L/R isoform 1 (PKLR)	153792131	1.0101	0.0018	C, Mi, ES, N	GluM, AP
Dihydrolipoamide branched chain transacylase E2 (DBT)	170172520	−1.0029	0.0000	MM	M
Glutaryl-CoA dehydrogenase (GCDH)	390190197	−1.0046	0.0000	MM	ACoAM, FAM, TM
Cytochrome P450 4A12A (CYP4A12A)	86198312	−1.0116	0.0001	EPR, MS	FAM
Ornithine carbamoyltransferase (OTC)	6679184	−1.0247	0.0000	MM, Mi	M, OM, RI
Glycine N-methyltransferase (GNMT)	6754026	−1.0490	0.0000	C	M, GM
Mitochondrial amidoxime-reducing component 1 (MARC1)	124486921	−1.0786	0.0218	Mi	ORP
Argininosuccinate synthase (ASS)	6996911	−1.0930	0.0120	C, EPR, Mi, N	APR, AB, OM
Tetratricopeptide repeat protein 36 (TTC36)	20336734	−1.1887	0.0201	U	U
4-aminobutyrate aminotransferase (ABAT)	37202121	−1.2385	0.0000	C, MM	AM
Acyl-coenzyme A oxidase 1 (ACOX1)	66793429	−1.2793	0.0000	C, N	FAM
Carboxylesterase 2A (CES2A)	19527178	−1.3778	0.0002	C, EPR	PG, XM
Carbamoyl-phosphate synthetase 2, aspartate transcarbamylase, and dihydroorotase (CAD)	51093867	−1.4093	0.0000	C	AAM, ARB
Carboxylesterase 1E (CES1E)	19526804	−1.4131	0.0000	C, EPR	XM
Betaine--homocysteine S-methyltransferase 1 (BHMT1)	7709990	−1.4143	0.0000	C, EPR, E	BM
Aldehyde dehydrogenase 1 family member L1 (ALDH1L1)	27532959	−1.4325	0.0000	C, N	FM
Sulfotransferase family 4A member 1 (SULT4A1)	313151233	−1.9839	0.0000	N	ALS, AMS
Major urinary protein 2 (MUP2)	530354677	−2.0297	0.0000	ES	T

Abbreviations: C, cytoplasm; CB, cytoplasmic bridges; CM, cellular membrane; CMT, cytoplasmic microtubule; E, exosome, ES, extracellular space; EPR, endoplasmic reticulum; MM, mitochondrial matrix; MS, microsome; Mi, mitochondrion; N, nucleus; PM, plasma membrane; U, unknown. AAM, amino acid metabolism; ACoAM, acyl-CoA metabolism; ALS, alcohol sulfotransferase; AM, aminobutyric acid metabolism; AMS, amine sulfotransferase; APR, acute phase response; ARB, arginine biosynthesis; BC, blood coagulation; BM, betaine metabolism; OM, ornithine metabolism; AP, apoptosis; BFCD, brown fat cell differentiation; CP, cellular proliferation; CR, cellular respiration; GBP, glutamine biosynthetic process; GM, glutathione metabolism; GluM, glucose metabolism; FAM, fatty acid metabolism; FM, folic acid metabolism; LM, lipid metabolism, LT, lipid transport; LS, lipid storage; M, metabolism; MO, motility; OM, ornithine metabolism; ORP, oxidation-reduction process; PF, protein folding; PG protein glycosylation; PRIS, positive regulation of insulin secretion; RCI, response to calcium ion; RERS, response to endoplasmic reticulum stress; RI, response to insulin; T, transporter; TM, tryptophan metabolism; XM, xenobiotic metabolism; U, unknown.

**Table 4:** List of upstream regulators activated and downregulated in liver tumors of TSOD mice detected by IPA.

Upstream Regulator	Molecule Type	Predicted Activation State	Activation z-Score	*p*-Value
NFE2L2	transcription regulator	Activated	3.110	5.8E-18
TRAP1	enzyme	Activated	2.975	5.66E-08
HNF1A	transcription regulator	Activated	2.795	8.18E-08
NR1I2	ligand-dependent nuclear receptor	Activated	2.470	1.19E-29
IL10RA	transmembrane receptor	Activated	2.350	0.0000
RICTOR	other	Activated	2.333	0.0012
CD3	complex	Activated	2.213	0.0301
TNF	cytokine	Activated	2.212	0.0188
IGF-1	growth factor	Activated	2.200	0.0313
VCAN	other	Activated	2	0.0311
PRKAA1	kinase	Inactivated	−2.121	0.0000
IL4	cytokine	Inactivated	−2.136	0.0169
WT1	transcription regulator	Inactivated	−2.200	0.0377
PRKAA2	kinase	Inactivated	−2.333	0.0000

Abbreviations: NFE2L2, nuclear factor, erythroid 2 like 2; TRAP1, TNF receptor associated protein 1; HNF1A, hepatocyte nuclear factor 1 homeobox A; NR1I2, nuclear receptor subfamily 1 group I member 2; IL10RA, interleukin 10 receptor subunit alpha; RICTOR, rapamycin-insensitive companion of mTOR; TNF, tumor necrosis factor; IGF-1, insulin like growth factor 1; VCAN, versican; PRKAA1, protein kinase AMP-activated catalytic subunit alpha 1; IL4, interleukin 4; WT1, Wilms tumor 1; PRKAA2, protein kinase AMP-activated catalytic subunit alpha 2.

**Table 5:** Immunohistochemical findings for liver tumors and liver tissues of TSOD and TSNO mice.

Proteins	Scores	Liver Tumors (TSOD)	NASH Liver Tissues (TSOD)	Control Liver Tissues (TSNO)	
mTOR					
	score 3+	39 (95%)	33 (100%)	10 (100%)	
	score 2+	0 (0%)	0 (0%)	0 (0%)	
	score 1+	2 (5%)	0 (0%)	0 (0%)	
	score 0	0 (0%)	0 (0%)	0 (0%)	
p-mTOR					
	score 3+	20 (49%) *	1 (3%)	0 (0%)	
	score 2+	3 (7%)	0 (0%)	0 (0%)	
	score 1+	13 (32%)	31 (94%)	10 (100%)	
	score 0	5 (12%)	1 (3%)	0 (0%)	
S6					
	score 3+	40 (97%)	33 (100%)	10 (100%)	
	score 2+	1 (3%)	0 (0%)	0 (0%)	
	score 1+	0%	0 (0%)	0 (0%)	
	score 0	0%	0 (0%)	0 (0%)	
p-S6					
	score 3+	17 (41%) *	3 (9%)	0 (0%)	
	score 2+	2 (5%)	3 (9%)	0 (0%)	
	score 1+	18 (44%)	24 (73%)	2 (20%)	
	score 0	4 (10%)	3 (9%)	8 (80%)	
FOXO3A (cytoplasmic)					
	score 3+	3 (7%)	1 (3%)	0 (0%)	
	score 2+	0 (0%)	0 (0%)	0 (0%)	
	score 1+	0 (0%)	0 (0%)	0 (0%)	
	score 0	38 (93%)	32 (97%)	10 (100%)	
p-FOXO3A (cytoplasmic)					
	score 3+	19 (46%) *	5 (15%)	0 (0%)	
	score 2+	5 (12%)	3 (9%)	0 (0%)	
	score 1+	16 (39%)	18 (55%)	5 (50%)	
	score 0	1 (2%)	7 (21%)	5 (50%)	

Score 0, negative staining; score 1+, positive staining in <25%; score 2+, positive staining in 25–50%; score 3+, positive staining in >50% of cells in the particular area. Data are numbers of positively stained mice livers or tumors (Incidence (%)). * *p* < 0.05 compared to control liver tissues of TSNO mice.

**Table 6:** Immunohistochemical findings for mTOR and p-mTOR in mouse model liver tumors and human HCCs.

Subjects-	mTOR				p-mTOR			
	score 3+	score 2+	score 1+	score 0	score 3+	score 2+	score 1+	score 0
Mouse models								
Liver tumors in TSOD mice	39 (95%)	0 (0%)	0 (0%)	2 (5%)	20 (49%)	3 (7%)	13 (32%)	5 (12%)
Liver tumors in CDAHFD-fed mice	33 (94%)	1 (3%)	1 (3%)	0 (0%)	1 (3%)	1 (3%)	23 (66%)	10 (28%)
Liver tumors in mice given DEN and PB	84 (89%)	7 (8%)	3 (3%)	0 (0%)	1 (1%)	5 (5%)	54 (58%)	34 (36%)
Human HCCs								
Metabolic syndrome/NASH-associated HCCs	30 (65%)	7 (15%)	9 (20%)	0 (0%)	29 (63%)	6 (13%)	11 (23%)	0 (0%)
virus-associated HCCs	18 (58%)	2 (7%)	1135 (%)	0 (0%)	2 (7%)	3 (10%)	18 (58%)	8 (25%)

Score 0, negative staining; score 1+, positive staining in <25%; score 2+, positive staining in 25–50%; score 3+, positive staining in >50% of cells in the particular area. Data are numbers of positively stained liver tumors (Incidence (%)).

## Supplementary Materials

The following are available online at http://www.mdpi.com/2072-6694/10/12/465/s1, Table S1: Incidence and multiplicity data for hepatocellular adenomas (HCAs), hepatocellular carcinomas (HCCs) and total liver tumors in TSOD and CDAHFD-fed C57BL/6J mice, Table S2: Clinicopathological characteristics of patients with HCC.
