# Supplementary material for: mTOR Activation in Liver Tumors Is Associated with Metabolic Syndrome and Non-Alcoholic Steatohepatitis in Both Mouse Models and Humans
Source: Cancers (Basel). 2018 Nov 22;10(12):465. doi: 10.3390/cancers10120465 (PMC6315895; doi:10.3390/cancers10120465)
Supplement: Supplementary file 1 [file cancers-10-00465-s001.pdf]

Supplementary Materials: mTOR Activation in Liver Tumors Is Associated with Metabolic Syndrome and Non-Alcoholic Steatohepatitis in Both Mouse Models and Humans

Takahiro Okuno, Anna Kakehashi, Naomi Ishii, Masaki Fujioka, Min Gi and Hideki Wanibuchi

Table S1. Incidences and multiplicities of total liver tumors, hepatocellular adenomas, and hepatocellular carcinomas in TSOD- and CDAHFD-fed C57BL/6J mice.

**Age**	**52 weeks**		**60 weeks**		**38 weeks**		**48 weeks**	
Animals	TSNO	TSOD	TSNO	TSOD	Control	CDAHFD	Control	CDAHFD
No. of animals [a)]	5	18	5	16	10	10	8	10
Liver tumors (HCC + HCA)								
Incidence (%)	1 (20)	11 (61)	0 (0)	13 (81) *	0	5 (50) *	0	8 (80) *
Multiplicity (No./mouse)	0.2 ± 0.4	1.0 ± 1.0	0.0 ± 0.0	1.8 ± 1.3 *	0.0 ± 0.0	1.2 ± 1.9	0.0 ± 0.0	2.2 ± 1.4 *
HCA								
Incidence (%)	0 (0)	8 (44)	0 (0)	8 (50)	0	5 (50) *	0	8 (80) *
Multiplicity (no./mouse)	0.0 ± 0.0	0.5 ± 0.6	0.0 ± 0.0	0.6 ± 0.6	0.0 ± 0.0	1.1 ± 1.7	0.0 ± 0.0	2.2 ± 1.4 *
HCC								
Incidence (%)	1 (20)	6 (33)	0 (0)	10 (63) *	0	1 (10)	0	0 (0)
Multiplicity (No./mouse)	0.2 ± 0.4	0.5 ± 0.9	0.0 ± 0.0	1.3 ± 1.3	0.0 ± 0.0	0.1 ± 0.3	0.0 ± 0.0	0.0 ± 0.0

HCC, hepatocellular carcinoma; HCA, hepatocellular adenoma; * $p < 0.05$, as compared to the control. [a] Final number of survived animals subjected to the analysis.

Table S2. Clinicopathological characteristics of patients with hepatocellular carcinomas.

	**Metabolic Syndrome/NASH-Associated HCCs**	**Virus-Associated HCCs**
age (years)	51–82 (mean 71.8)	21–83 (mean 64.6)
sex		
male	37 (80%)	17 (55%)
female	9 (20%)	14 (45%)
complications		
obesity	24 (52%)	0
diabetes	32 (70%)	0
dyslipidemia	19 (41%)	0
hypertension	38 (83%)	0
hepatitis B virus infection	0	11 (35%)
hepatitis C virus infection	0	20 (65%)
differentiation of HCC		
well-differentiated	8 (17%)	6 (19%)
moderately differentiated	27 (59%)	19 (62%)
poorly differentiated	11 (24%)	6 (19%)
fibrosis of liver		
no fibrosis	1 (2%)	0
fibrous portal expansion	16 (36%)	9 (29%)
bridging fibrosis	15 (33%)	17 (55%)
cirrhosis	13 (29%)	5 (16%)
NASH histology		
confirmed by biopsy	2 (4%)	-
confirmed with surgical specimens	15 (33%)	-
